# The Role of Preoperative Dynamic Contrast-enhanced 3.0-T MR Imaging in Predicting Early Recurrence in Patients With Early-Stage Hepatocellular Carcinomas After Curative Resection

**DOI:** 10.3389/fonc.2019.01336

**Published:** 2019-11-28

**Authors:** Linqi Zhang, Sichi Kuang, Jingbiao Chen, Yao Zhang, Binliang Zhao, Hao Peng, Yuanqiang Xiao, Kathryn Fowler, Jin Wang, Claude B. Sirlin

**Affiliations:** ^1^Department of Radiology, The Third Affiliated Hospital of Sun Yat-sen University, Guangzhou, China; ^2^Department of Nuclear Medicine, Affiliated Cancer Hospital and Institute of Guangzhou Medical University, Guangzhou, China; ^3^Liver Imaging Group, Department of Radiology, University of California, San Diego, La Jolla, CA, United States

**Keywords:** magnetic resonance imaging, hepatocellular carcinoma, recurrence, LI-RADS, liver resection

## Abstract

**Objectives:** Liver resection is potentially curative for early-stage hepatocellular carcinoma (eHCC) in patients with well-preserved liver function. The prognosis of these patients after resection is still unsatisfactory because of frequent early recurrence (ER). Therefore, we investigated the role of preoperative dynamic contrast-enhanced 3.0-T MR imaging in predicting ER of eHCC after curative resection.

**Methods** From May 2014 to October 2017, we retrospectively analyzed 82 patients with eHCC who underwent dynamic MR imaging and subsequently underwent curative resection. Liver Imaging Reporting and Data System (LI-RADS) v2018 major and ancillary imaging features, as well as two non-LI-RADS MR imaging features (irregular tumor margin and tumor number), were evaluated. A multivariate Cox regression analysis was used to identify independent predictors, and two models (preoperative and postoperative prediction models) were developed.

**Results** ER was observed in 25 patients (25/82, 30.5%). In the univariate analyses, preoperative alpha-fetoprotein (AFP) level >200 ng/ml, three MR imaging features (multifocal tumors, corona enhancement, and irregular tumor margin), and microvascular invasion (MVI) were associated with ER. In the multivariate analysis, corona enhancement (hazard ratio [HR]: 2.970; *p* = 0.013) and irregular tumor margin (HR: 2.377; *p* = 0.048) were independent predictors in the preoperative prediction model, and preoperative AFP level >200 ng/ml (HR: 2.493; *p* = 0.044) plus corona enhancement (HR: 3.046; *p* = 0.014) were independent predictors in the postoperative prediction model (microvascular invasion [MVI] was not; *p* = 0.061). When combined with both predictors, the specificity for ER in the preoperative prediction model was 98.2% (56/57), which was comparable to that of the postoperative prediction model [96.7% (55/57)].

**Conclusions** Our results demonstrated that preoperative MR imaging features (corona enhancement and irregular tumor margin) have the potential to preoperatively identify high-risk ER patients with eHCC, with a specificity >90%.

## Introduction

Hepatocellular carcinoma (HCC) is the fifth most common cancer and is the third leading cause of cancer-related deaths worldwide (1). Recently, the frequency of detection of early-stage hepatocellular carcinoma (eHCC, defined as up to three nodules with diameter ≤ 3 cm) has increased due to the screening of high-risk populations and advances in imaging techniques (2). Liver resection is potentially curative for these patients with well-preserved liver function. However, early recurrence (ER), defined as intrahepatic, regional, or systemic recurrence within 12 months after resection, occurs in ~20–40% of eHCC patients and is the leading cause of postoperative death (3, 4). Thus, there is a need to identify high-risk ER patients with eHCC so that a more aggressive surgery (such as liver transplantation or a wider extent of resection) as well as a strict follow-up protocol can be established. Previous studies have suggested that clinicopathological variables, including the presence of microvascular invasion (MVI), worse histological differentiation, microsatellite nodules, alpha-fetoprotein (AFP) level, and tumor size, were significant predictors for ER patients with eHCC after curative resection, but controversy exists as to which of these are more important, and some of these predictors can only be evaluated with postoperative pathological examination (5–7).

Currently, several studies have attempted to evaluate preoperative MR imaging features (such as tumor size, irregular tumor margin, and peritumoral parenchymal enhancement) in predicting MVI and ER of HCC (8–10). The Liver Imaging Reporting and Data System (LI-RADS) is an imaging system for standardized interpretation, reporting, and data collection for imaging examinations in patients at risk for HCC, which was launched in 2011, with recent updates in 2018 and integration into AASLD clinical practice guidance (2, 11). The aim of LI-RADS is to help radiologists categorize liver imaging findings and facilitate communication between radiologists and other physicians by using a common terminology. The system addresses the full spectrum of liver lesions and pseudolesions with a 5-point scale reflecting the relative likelihood of HCC, from LR-1 (definitely benign) to LR-5 (definitely HCC) and LR-M. Although LI-RADS was conceived as a diagnostic system, LI-RADS imaging features (12–14) as well as some non-LI-RADS imaging features, such as tumor number (6) and irregular tumor margins (10, 15, 16), may provide prognostic information. However, to the best of our knowledge, few studies have attempted to identify major and ancillary imaging features predicting ER in patients with eHCC after curative resection as determined by LI-RADS. Accordingly, the purpose of the present study was to identify the preoperative predictors (including MR imaging features and tumor marker) for ER and establish a preoperative prediction model in patients undergoing curative resection of eHCC.

## Materials and Methods

### Patients

**Figure 1:**
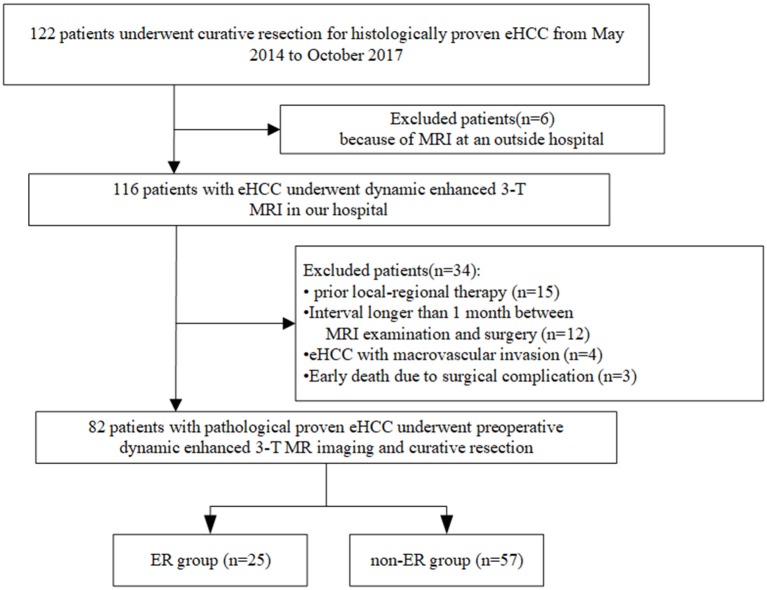
The workflow of patient selection for this study.

From May 2014 to October 2017, we undertook a single-center retrospective study, which was approved by our institutional review board, and the requirement to obtain written informed consent was waived. We reviewed information from our pathology and radiology database. The workflow of patient selection for this study is detailed in Figure 1. A total of 122 patients with a pathological diagnosis of eHCC who underwent dynamic enhanced 3.0-T MR imaging and subsequently underwent curative resection (R0, defined as a histological removal of all tumors and negative margin) were included. We excluded 40 patients for the following reasons: prior local-regional therapy (*n* = 15); the interval between MR examination and surgery was longer than 1 month (*n* = 12); MR imaging was performed at an outside hospital (*n* = 6); HCC with macrovascular invasion was observed on MR imaging (*n* = 4); and surgical complications resulted in an early death (*n* = 3). Finally, a total of 82 patients with eHCC were enrolled. These patients were divided into two groups: patients who suffered from recurrence within 1 year after surgery (the ER group) and patients who were disease-free for more than 1 year (the non-ER group).

### MRI Protocol

All patients were scanned in supine position on a 3.0-T whole-body MR scanner (Discovery MR750, GE Healthcare, Milwaukee, WI) with an eight-channel phased-array coil centered over the abdomen. All patients had fasted for at least 4 h before examination. Precontrast pulse sequences included breath-hold coronal fast imaging employing steady-state acquisition (FIESTA), breath-hold coronal single-shot fast spin echo (SSFSE), respiratory-triggered axial T2-weighted fast spin echo (FSE), breath-hold two-dimensional dual-echo T1-weighted gradient-recalled-echo images at ~1.15 (opposed phase) and 2.3 (in phase) ms, and respiratory-triggered axial diffusion-weighted spin-echo echo-planar imaging with 2 b values (b = 0 and 800 s/mm^2^). DWI was performed using a respiratory-triggered single-shot spin echo echoplanar imaging sequence in the transverse plane before contrast-enhanced imaging. The acquisition of the DWI sequence with multiple b values typically took between 4 and 7 min. Acquisition matrix = 128 × 128, field of view = 38 × 30 cm, slice thickness = 5 mm, and slice gap = 1 mm. Afterwards, breath-hold 3D T1W gradient-recalled-echo imaging (liver acquisition with volume acceleration [LAVA]) was performed before and at multiple time points dynamically after injection of extracellular contrast media (ECCM, various formulations, 0.1 mmol of gadolinium per kilogram of body weight, *n* = 32 patients), or gadobenate dimeglumine (MultiHance, Bracco Diagnostics, Milan, Italy; 0.1 mmol of gadolinium per kilogram of body weight, *n* = 50 patients), followed by a 20 mL saline flush at 2 mL/s. A dual arterial phase (AP) sequence was initiated 15–20 s after the contrast media arrived at the distal thoracic aorta using bolus triggering, a dual portal venous phase (PVP) was acquired at 1 min after contrast injection, and a delayed phase (DP) was acquired at 3 min. Additional hepatobiliary phase images were obtained 60–120 min after injection of gadobenate dimeglumine.

### Image Analysis

Image analysis was performed by two abdominal radiologists (with 5 years [initials withheld during submission] and 24 years [initials withheld during submission] of experience in liver MR imaging) who were unaware of the clinical, laboratory, pathologic, and follow-up results. Reviewers independently evaluated the imaging features for each selected observation as defined in LI-RADS v2018 (2). A LI-RADS category was assigned to each selected observation according to the LI-RADS major features and ancillary features. We also evaluated two non-LI-RADS imaging features, irregular tumor margin (10) and tumor number (6), which can predict ER in HCC, as proposed in several previous studies. Irregular tumor margin was defined as non-nodular tumor in all imaging planes (17). Disagreements were resolved by consensus.

### Pathological Analysis

All original hepatic specimens were reviewed by a hepatic pathologist with more than 20 years of experience in hepatic pathology who was blinded to the imaging findings. The number and size of eHCCs, the extent of liver fibrosis and tumor capsule formation, MVI, and the microscopic margins were recorded from the pathological examination reports. Additional immunohistochemical staining was carried out for cytokeratin 19 (CK19). The liver fibrosis stage of the non-tumor-bearing liver parenchyma was assessed from the liver specimens using the METAVIR staging system (18). The degree of tumor differentiation was categorized as well/moderately/poorly differentiated according to the Lauwers classification (19). When different grades coexisted within a tumor, the predominant grade of the tumor was used (>50%). Tumor capsule formation was considered positive when the capsule was found along at least two thirds of the tumor margin, regardless of the presence of microscopic capsular or extracapsular invasion (20). MVI was defined as observed only on microscopy (21). Immunoreactivity for CK19 was classified as negative (<5% of tumor cells) or positive (>5% of tumor cells) (22).

### Follow-Up

After resection, all 82 patients were followed up for at least 1 year. Postoperative follow-up included clinical examination, chest radiography, biochemical liver function tests, and serum levels of serum α-fetoprotein (AFP) performed every 1 month during the first year after hepatic resection and then every 2–3 months afterwards. In addition, contrast-enhanced MRI was performed every 3 months. Recurrence was diagnosed by pathological examination after rehepatectomy or appropriate imaging characteristics according to criteria of the guidelines by the American Association for the Study of Liver Diseases (23).

### Statistical Analysis

Continuous variables are expressed as the means ± standard deviations (SD) and were compared using Student's *t*-test. Categorical variables were compared using the chi-square test. Variables were dichotomized using a cutoff of 2 cm in tumor size (24) and 200 ng/ml in AFP level (3), as previously proposed. Recurrence-free survival (RFS) was calculated by the Kaplan–Meier method, and differences in survival between the ER and non-ER groups were compared using the log-rank test (univariate analysis). Variables with *P* < 0.05 on univariate analysis were subjected to Cox regression multivariate analysis to identify independent predictors of ER, and then a preoperative model (based on clinical and radiological factors) and a postoperative prediction model (based on clinical, radiological, and pathological factors) were developed. The results of these models are expressed as the hazard ratio (with 95% CI). The sensitivity, specificity, positive predictive value (PPV), and negative predictive value (NPV) of the multivariate prediction models identified above were estimated for ER, and the diagnostic performance was evaluated by ROC. The area under the curve (AUC) was calculated for each model and compared against each other using the DeLong method to determine if a significant difference was present. *P* < 0.05 were considered statistically significant. All statistical tests were performed using SPSS Statistics 17.0 (SPSS Inc., Chicago, IL, USA) software.

## Results

### Clinical Characteristics and Recurrence

The baseline demographic characteristics of the 82 patients (74 males and 8 females; mean age: 51.9 ± 8.0 years; range: 22–78 years) with eHCC according to ER are summarized in Table 1. Recurrence was observed in 31 patients: ER in 25 patients (25/82, 30.5%) and later recurrence (>1 year) in 6 patients (14, 16, 21, 30, 33, and 42 months). The most common site for ER was intrahepatic (*n* = 24), followed by extrahepatic (lung, *n* = 1). All sites for later recurrence were intrahepatic (*n* = 6). The mean recurrence time for the ER group was 7.4 ± 2.7 months (range: 2–12 months) after hepatic resection. In the non-ER group, the mean follow-up was 30.8 ± 10.3 months (median: 32 months; range: 12–52 months). The median serum AFP was 24.2 ng/mL (1–990 ng/mL), and a preoperative AFP level >200 ng/ml was more frequently observed in the ER group than in the non-ER group (13 of 25 [52%] vs. 16 of 57 [28.1%], respectively; *p* = 0.034). No variables showed significant differences between the two groups in other clinical characteristics, such as age, sex, or liver function tests.

**Table 1:** Patient characteristics according to ER.

**Variable**	**Total (*n* = 82)**	**ER (*n* = 25)**	**Non-ER (*n* = 57)**	***p*-value**
**Demographics**				
Mean age (y)	51.3 ± 10.4 (22–78)	51.1 ± 11.3 (22–73)	51.9 ± 8.0 (40–78)	0.808
Sex				
Male	74 (90.2%)	23 (92%)	51 (89.5%)	0.357
Female	8 (9.8%)	2 (8%)	6 (10.5%)	
Etiology				
HBV	79 (96.3%)	23 (92%)	56 (98.2%)	0.219
HCV	3 (3.7%)	2 (8%)	1 (1.8%)	
Child-Pugh				
A	81 (98.8%)	24 (96%)	57 (100%)	0.305
B	1 (1.2%)	1 (4%)	0 (–)	
Serum AFP (ng/ml)				
≤ 200	53 (64.6%)	12 (48%)	41 (71.9%)	0.034
>200	29 (35.4%)	13 (52%)	16 (28.1%)	
LI-RADS				
LR-3 (8) +				
LR-4 (7)	15 (18.3%)	4 (16%)	11 (19.3%)	0.493
LR-5 (65) + LR-M (2)	67 (81.7%)	21 (84%)	46 (80.7%)	
DFS time	23.7 ± 13.9 (2–52)	7.4 ± 2.8 (2–12)	30.8 ± 10.3 (2–52)	–
**MRI finding**				
Tumor size (cm)	2.1 ± 0.6 (0.5–3)	2.2 ± 0.8 (0.8–3)	2.1 ± 0.5 (0.5–3)	
Tumor number				
1	68 (82.9%)	18 (72%)	50 (87.7%)	0.080
>1	14 (17.1%)	7 (28%)	7 (12.3%)	
Irregular margin				
Present	25 (30.5%)	14 (56%)	11 (19.3%)	0.001
Absent	57 (69.5%)	11 (44%)	46 (80.7%)	
**LI-RADS major features**				
Tumor size				
≤ 2 cm	31 (37.8%)	9 (36%)	22 (38.6%)	0.513
>2 cm	51 (62.2%)	16 (64%)	35 (61.4%)	
APHE				
Present	76 (92.7%)	23 (92%)	53 (93.0%)	0.597
Absent	6 (7.3%)	2 (8%)	4 (7.0%)	
Washout				
Present	68 (82.9%)	20 (80%)	48 (84.2%)	0.431
Absent	14 (17.1%)	5 (20%)	9 (15.8%)	
Enhancing Capsule				
Present	51 (62.2%)	14 (56%)	37 (64.9%)	0.300
Absent	31 (37.8%)	11 (44%)	20 (35.1%)	
**LI-RADS ancillary features**				
Mosaic architecture				
Present	28 (34.1%)	10 (40%)	18 (31.6%)	0.310
Absent	54 (65.9%)	15 (60%)	39 (68.4%)	
Nodule-in-nodule architecture				
Present	80 (97.6%)	25 (100%)	55 (96.5%)	0.481
Absent	2 (2.4%)	0 (0%)	2 (3.5%)	
Fat in mass				
Present	23 (28.0%)	8 (32%)	15 (26.3%)	0.392
Absent	59 (72.0%)	17 (68%)	42 (73.7%)	
Blood products				
Present	79 (96.3%)	24 (96%)	55 (96.5%)	0.670
Absent	3 (3.7%)	1 (4%)	2 (3.5%)	
Corona enhancement				
Present	20 (24.4%)	10 (40%)	10 (17.5%)	0.031
Absent	62 (75.6%)	15 (60%)	47 (82.5%)	
Restricted diffusion				
Present	75 (91.5%)	25 (100%)	50 (87.7%)	0.070
Absent	7 (8.5%)	0	7 (12.3%)	
**Pathologic factors**				
Differentiation				
Well + moderate	60 (73.2%)	20 (80%)	40 (70.2%)	0.260
Poor	22 (26.8%)	5 (20%)	17 (29.8%)	
Tumor capsule				
Present	59 (72%)	17 (68%)	42 (73.7%)	0.392
Absent	23 (28%)	8 (32%)	15 (26.3%)	
CK 19				
Positive	11 (13.4%)	4 (16%)	7 (12.3%)	0.446
Negative	71 (86.6%)	21 (84%)	50 (87.7%)	
MVI				
Absent	64 (78.0%)	15 (60%)	49 (86.0%)	0.012
Present	18 (22.0%)	10 (40%)	8 (14.0%)	
Fibrosis stage				
Early (F0–F2)	19 (%)	6 (24%)	13 (22.8%)	0.558
Advanced (F3–F4)	63 (%)	19 (76%)	44 (77.2%)	

*AFP, alpha-fetoprotein; APHE, arterial phase hyperenhancement; MVI, microvascular invasion; CK19, cytokeratin 19*.

### MR Imaging Features

A total of 99 eHCCs were detected in the 82 patients, 11 patients had two lesions each, three patients had three lesions each, and the remaining 68 patients had one lesion each. The mean size of the largest eHCC was 2.1 ± 0.6 cm (range, 0.5–3 cm), and in 51 patients (62.2%), the largest tumor size was >2 cm; only 2 patients (2.4%) had a solitary tumor size ≤ 1 cm. On the basis of LI-RADS v2018, patients with eHCC were assigned as LR-3 in 8 patients (9.8%), LR-4 in 7 patients (8.5%), LR-5 in 65 patients (79.3%), and LR-M in 2 patients (2.4%). The ER rate was higher for HCCs categorized as LR-5 or LR-M (21/67, 31.3%) than for those categorized LR-3 or LR-4 (4/15, 24.4%), but this difference was not statistically significant (*p* = 0.493). Regarding MR imaging features, 25 patients (30.5%) had an irregular margin, and 20 patients (24.4%) showed corona enhancement. Irregular margins were more frequently observed in the ER group than in the non-ER group (14 of 25 [56%] vs. 11 of 57 [19.3%], respectively; *p* = 0.001). In addition, corona enhancement was more frequently observed in the ER group than in the non-ER group (10 of 25 [40%] vs. 10 of 57 [17.5%], respectively; *p* = 0.031). With regard to LI-RADS major features or other LI-RADS ancillary features, there were no statistically significant differences between the groups with and without ER.

### Pathological Findings

Differentiation according to the Lauwers classification resulted in 60 well or moderately differentiated tumors (73.2%) and 22 poorly differentiated (26.8%) tumors, but there was no statistically significant difference between the groups with and without ER (*p* = 0.26). There was evidence of MVI in 18 (22%) of the 82 patients. MVI was more frequently observed in the ER group than in the non-ER group (10 of 25 [40%] vs. 8 of 57 [14.0%], respectively; *p* = 0.012). With regard to cirrhosis (*p* = 0.558), tumor capsule (*p* = 0.392), or CK19 (*p* = 0.446), there were no statistically significant differences between the groups with and without ER.

### Univariate and Multivariate Analysis Factors Predictive of ER

**Figure 2:**
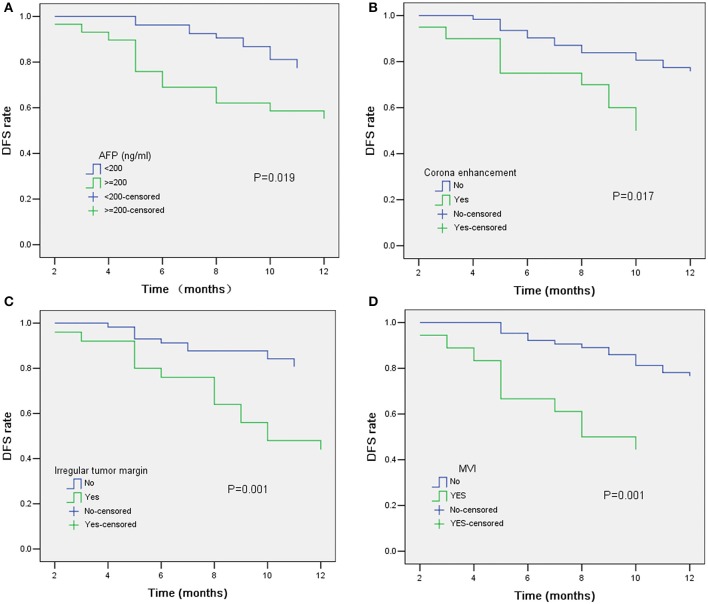
Kaplan–Meier curves for RFS showing significant difference is seen between patients classified as: **(A)** AFP ≤ 200 ng/ml and AFP > 200 ng/ml (log-rank test, *p* = 0.019); **(B)** corona enhancement (+) and corona enhancement (–) (log-rank test, *p* = 0.017); **(C)** irregular tumor margin (+) and irregular tumor margin (–) (log-rank test, *p* = 0.001); **(D)** MVI (+) and MVI (–) (log-rank test, *p* = 0.001).

**Figure 3:**
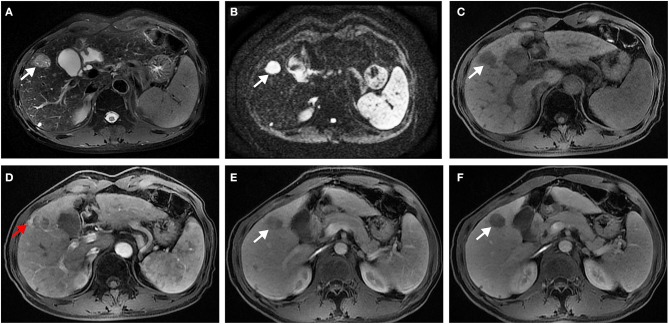
Surgically confirmed moderately differentiated HCC with AFP = 245 ng/mL. MR imaging showed a hepatic nodule (2.6 cm, arrow) in S4 with mild-moderate T2 hyperintensity **(A)** and restricted diffusion **(B)**. The nodule is hypointense on precontrast T1WI **(C)** and shows corona enhancement (red arrow) in late arterial phase **(D)**, and washout appearance on portal venous **(E)**, and delayed **(F)** phase. Histology confirmed MVI (+). This patient recurred 10 months after resection.

**Figure 4:**
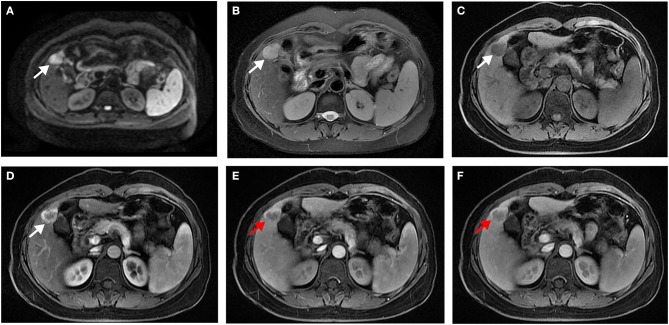
Surgically confirmed moderately differentiated HCC with AFP = 520 ng/mL. MR imaging showed a hepatic nodule (2.8 cm, arrow) in S4/5 with restricted diffusion **(A)** and mild-moderate T2 hyperintensity **(B)**. The nodule is hypointense on precontrast T1WI **(C)** and shows hyperenhancement in late arterial phase **(D)**, and irregular tumor margin (red arrow) on portal venous **(E)**, and delayed **(F)** phase. Histology confirmed MVI (–). This patient recurred 5 months after resection.

**Figure 5:**
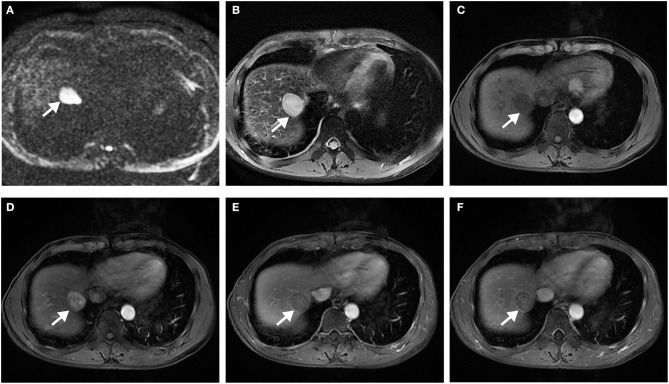
Surgically confirmed moderately differentiated HCC with AFP = 3 ng/mL. MR imaging showed a hepatic nodule (2.9 cm, arrow) in S7/8 with restricted diffusion **(A)** and mild-moderate T2 hyperintensity **(B)**. The nodule is hypointense on precontrast T1WI **(C)** and shows hyperenhancement, without corona enhancement in late arterial phase **(D)**, smooth tumor margin in portal venous **(E)**, and delayed **(F)** phase. Histology confirmed MVI (+). This patient did not have any recurrence after resection during the 37 months follow-up period.

In univariate analysis (Table 2), preoperative AFP level >200 ng/ml (*p* = 0.019), Figure 2A, three MR imaging features (multifocal tumors [*p* = 0.05], corona enhancement [*p* = 0.017], Figure 2B, and irregular tumor margin [*p* = 0.001]), Figure 2C, and one pathological factor (MVI [*p* = 0.001], Figure 2D) were associated with ER. In the multivariate analysis (Table 3), only corona enhancement (hazard ratio [HR]: 2.970; *p* = 0.013; Figure 3) and irregular tumor margin (HR: 2.377; *p* = 0.048; Figure 4) were independent predictors in the preoperative prediction model, and preoperative AFP level >200 ng/ml (HR: 2.493; *p* = 0.044) and corona enhancement (HR: 3.046; *p* = 0.014) were independent predictors in the postoperative prediction model. Figure 5 showed a patient with smooth tumor margin and absent of corona enhancement on MRI and did not have any recurrence after resection (follow up 37 months).

**Table 2:** Univariate analysis of preoperative and pathologic predictors for early recurrence of eHCC patients (*n* = 82).

**Variable**	**HR**	**95% CI**	***p*-value**
**Preoperative factors**			
AFP >200 ng/ml	2.498	1.138–5.483	0.019^*^
Multifocal tumors	2.304	1.095–5.535	0.05^*^
APHE	0.826	0.195–3.505	0.792
Tumor size ≥2 cm	1.126	0.498–2.549	0.772
Washout	1.703	0.510–5.692	0.374
Enhancing Capsule	0.765	0.347–1.684	0.497
Mosaic architecture	1.435	0.644–3.197	0.367
Nodule-in-nodule architecture (absent)	0.845	0.578–2.214	0.385
Fat in mass (absent)	1.288	0.556–2.986	0.472
Blood products	1.223	0.165–9.045	0.841
Corona enhancement	2.524	1.132–5.628	0.017^*^
Irregular tumor margin	3.649	1.651–8.063	0.001^*^
Restricted diffusion	0.924	0.718–2.415	0.091
**Postoperative pathologic factors**			
High grade (G3 or G4)	1.666	0.625–4.442	0.212
CK 19 (positive)	1.301	0.447–3.793	0.623
Tumor capsule	0.655	0.289–1.482	0.298
MVI	2.338	1.060–5.517	0.001^*^
Fibrosis stage (F3–F4)	0.920	0.367–2.304	0.856

*HR, hazard ratio; CI, confidence interval; AFP, alpha-fetoprotein; APHE, arterial phase hyperenhancement; MVI, microvascular invasion; CK19, cytokeratin 19*.

* *Statistically significant results from univariate analysis*.

**Table 3:** Multivariate analysis of preoperative and postoperative independent predictors for early recurrence of eHCC patients (*n* = 82).

**Variable**	**Pre-operative model**			**Post-operative model**		
	**HR**	**95% CI**	***p*-value**	**HR**	**95% CI**	***p*-value**
AFP >200 ng/ml	2.179	0.923–5.143	0.075	2.493	1.026–6.060	0.044^*^
Multifocal tumors	2.385	0.963–5.906	0.060	2.192	0.879–5.463	0.092
Corona enhancement	2.970	1.263–6.982	0.013^*^	3.046	1.256–7.386	0.014^*^
Irregular tumor margin	2.377	1.009–5.599	0.048^*^	1.867	0.760–4.586	0.173
MVI	–	–	–	2.285	0.964–5.418	0.061

*HR, hazard ratio; CI, confidence interval; AFP, alpha-fetoprotein; MVI, microvascular invasion*.

* *Statistically significant results from multivariate analysis*.

The diagnostic performance of the preoperative and postoperative prediction models are summarized in Table 4. Matching at least one predictor, the sensitivity and specificity for ER in the preoperative prediction model were 68 and 68.4%, respectively and those in the postoperative prediction model were 76 and 57.9%, respectively. The area under the curve (AUC) in the preoperative prediction model was 0.682 (95% confidence interval [CI], 0.55–0.81), slightly higher than that in the postoperative prediction model (0.669, 95% CI, 0.554–0.794), but this was not statistically significant (*p* = 0.581). Matching two predictors led to a higher specificity both in the preoperative (98.2%) and postoperative (96.5%) prediction models; however, sensitivity dropped markedly in both the preoperative (28%) and postoperative (16%) prediction models. The AUC in the preoperative prediction model was 0.612 (95% CI, 0.555–0.809), which was higher than that in the postoperative prediction model (0.534, 95% CI, 0.395–0.673), but this was also not statistically significant (*p* = 0.314).

**Table 4:** Prognostic performance of preoperative and postoperative prediction models.

**Models**	**Sensitivity (%)**	**Specificity (%)**	**PPV (%)**	**NPV (%)**	**Accuracy (%)**	**AUROC**
	**N/D**	**N/D**	**N/D**	**N/D**	**N/D**	
	**[95% CI]**	**[95% CI]**	**[95% CI]**			**[95% CI]**
**Matching at least one predictor**						
Preoperative model	68 (17/25) [46.4–84.3]	68.4 (39/57) [54.6–79.7]	48.6 (17/35) [31.7–65.7]	83.0 (39/47) [68.7–91.9]	68.3 (56/82)	0.682 [0.555–0.809]
Postoperative model	76 (19/25) [54.4–89.8]	57.9 (33/57) [44.1–70.6]	44.2 (19/43) [26.0–59.9]	84.6 (33/39) [68.8–93.6]	63.4 (52/82)	0.669 [0.544–0.794]
**Combined with two predictors**						
Preoperative model	28 (7/25) [12.9–49.6]	98.2 (56/57) [89.4–99.9]	87.5 (7/8) [46.7–99.3]	75.7 (56/74) [64.1–84.6]	76.8 (63/82)	0.602 [0.461–0.744]
Postoperative model	16 (4/25) [5.3–36.9]	96.7 (55/57) [86.8–99.4]	66.7 (4/6) [24.1–94.0]	72.4 (55/76) [60.7–81.7]	72.0 (59/82)	0.534 [0.395–0.673]

*AUC, the area under the curve; CI, confidence interval*.

## Discussion

In this study, we attempted to investigate the role of preoperative tumor markers and MR imaging features (focused on LI-RADS v2018 major and ancillary features, as well as some other validated non-LI-RADS imaging features) in predicting ER in eHCC after curative resection. The results showed that tumor markers (AFP level >200 ng/ml), one LI-RADS v2018 ancillary feature (corona enhancement), and two non-LI-RADS MR imaging features (multifocality and irregular tumor margin) were associated with ER, but only corona enhancement and irregular tumor margin were independent predictors in the preoperative prediction model. Various imaging features (such as tumor size, irregular margin, satellite nodule, peritumoral enhancement) have been investigated previously in predicting MVI, ER, and survival in patients with HCC (8–10, 25) but with ambiguous definitions, and studies have rarely focused on the eHCC group. Corona enhancement is an LI-RADS ancillary feature favoring malignancy, which is defined as periobservational enhancement on the late arterial phase or early portal venous phase outside the tumor border and becomes isointense with normal liver parenchyma at the subsequent dynamic phases (2). Based on previous studies (17, 26), it is conceivable that corona enhancement is involved in tumors, yielding microdissemination and then causing ER. Several studies (10, 15, 17) also reported that irregular tumor margin is an important predictor of invasive gross type and tumor recurrence in HCC. Ariizumi et al. (15) found that irregular margin tumor margin had 69.5% (41/59) accuracy in predicting tumor recurrence in HCC. In general, MR imaging scanners with high resolution have a higher sensitivity in distinguishing smooth margins of eHCC from irregular margins than contrast-enhanced CT scanners (27, 28). If an irregular tumor margin is confirmed in large-scale samples to have prognostic importance, then this feature may need to be added to the LI-RADS lexicon so that its definition can be standardized.

The influence of clinicopathological factors on outcomes after resection for eHCC has been well-established in a previous study (3–7, 9). In our series, tumor markers (AFP level >200 ng/ml) and corona enhancement were independent predictors in the postoperative prediction model according to multivariate analysis. It was interesting to note that MVI was not an independent predictor of ER in the postoperative prediction model, although it was a significant predictor in the univariate analysis. The presence of MVI is reported to be ~17–33% (4, 5, 24) in eHCC, and similar as these studies, MVI occurred in 22% of patients in our series. The significance of MVI as a predictor of long-term survival in patients with eHCC is still controversial. Several studies have reported that the presence of MVI was one of the most important risk factors related to tumor recurrence in eHCC (3, 29). Conversely, in another study with 1,109 patients with solitary eHCC from six major international hepatobiliary centers, MVI was not affected by long-term survival (24); our study is in accord with this study. The discrepancy between these results may be attributed to the heterogeneity of the patient population evaluated, the low proportion of MVI in eHCC, and further prospective cohort studies should be undertaken to validate our results. A validated biomarker of HCC, serum AFP, has been correlated with a high rate of ER and poor prognosis (3, 7, 30, 31). Mild to moderate elevation in AFP is sometimes found in patients with hepatitis or cirrhosis, but severe elevation of AFP to levels of 200 ng/mL or more is likely to be caused by HCC with high malignant potential (3). While our univariate results were consistent with those of previous studies, AFP was an independent predictor for ER in postoperative prediction models that incorporated corona enhancement.

When matching at least one predictor, the sensitivity and specificity for ER in the preoperative prediction model were 68 and 68.4%, respectively, and those in the postoperative prediction model were 76 and 57.9%, respectively. The prognostic performance of the preoperative prediction model was slightly higher than that of the postoperative prediction model (AUC, 0.682 vs. 0.669, but this was also not statistically significant). Matching two predictors led to higher specificities both in the preoperative (98.2%) and postoperative (96.5%) prediction models; however, the sensitivities dropped markedly in both the preoperative (28%) and postoperative (16%) prediction models. It seems that the preoperative prediction model was not inferior to the postoperative prediction model in predicting ER. We suggest that the combination of independent MR imaging features has a synergistic effect in predicting ER of eHCC preoperatively compared to each independent MR imaging feature alone.

Our study had several limitations. First, our study was limited by its retrospective design, which may have introduced selection bias. The sample size was not large, and the observation period was relatively short because 3.0-T MR imaging scanners have been in clinical use in our hospital since only May 2014. Second, the impact of the predictor on survival after resection was not analyzed because there were fewer patient deaths (only 3) related to recurrence of HCC during the study period. Further studies with prospectively randomized populations are warranted to confirm these promising results and hope to establish a better preoperative prediction model with higher sensitivity.

In conclusion, a combination of two preoperative MR imaging features (corona enhancement and irregular tumor margin) has the potential to preoperatively identify high-risk ER patients with eHCC, with a specificity >90%. Patients with independent MR imaging features, liver transplantation or a wider extent of resection may be considered.

## Data Availability Statement

The datasets generated for this study are available on request to the corresponding author.

## Ethics Statement

The current study was approved by the Institutional Ethics Committee of the Third Affiliated Hospital of Sun Yat-sen University (No. 2019-02-163-01), and the need for signed informed consent was waived.

## Author Contributions

LZ and SK participated in the design of the study and drafted the manuscript. JC and YZ collected the patients' data. BZ and YX interpreted the MRI images. HP processed the figures. JW conceived the study and supervised the project. KF and CS revised the manuscript. All authors read and approved the final version of the manuscript.

### Conflict of Interest

The authors declare that the research was conducted in the absence of any commercial or financial relationships that could be construed as a potential conflict of interest.
